# Foreign Summary

**Published:** 1840-02-22

**Authors:** 


### FOREIGN SUMMARY.

*Abstract of a Paper on Ulcerations of the Cervix Uteri, and on the Abuse of the Speculum Uteri, by* C. M. Gibert, M. D., Physician to the Hôpital de Lourcine. By Fleetwood Churchill, M. D.—This is an able paper, appearing at a very suitable time, when the assistance derived from the use of the speculum, in treating uterine diseases, is exaggerated far beyond the experience of practical men. The author commences by objecting to the practice of attributing the delicacy and disorders of females, leading an indolent life, to some derangement of the uterine function. "To place the seat of most of the disorders of females in the uterus, as some practitioners of our days have not hesitated to do, is to dress up an old error in the garb of improvement. But to treat actively, and by means more or less severe, all the changes in the cervix uteri, however slight and superficial they may be, is more than erroneous,—it is criminal.

"This fault, however, has become so common, that, strictly speaking, the invention of the speculum (which led to it) must be regarded equally injurious as useful, if we consider the numerous abuses into which certain practitioners have been led by the constant use of the instrument.

"It is not denied that the diagnosis of venereal diseases has acquired much precision from the employment of the speculum, although much incertitude remains as to the real importance of certain lesions even when exposed to view.

"But I hesitate not to affirm, for my part, that certain alterations, altogether insignificant, and having no relation to the symptoms exhibited by the patient, have been many times regarded as the source of these symptoms. I have observed many women who complained of abdominal pains, whites, and other symptoms which might lead one to attribute them to derangements of the uterus, without there being any appreciable lesion of that organ. In other cases, certain changes were discovered by the aid of the speculum, which had given rise to no symptoms at all. This counter-proof places, beyond all doubt, the innocuous nature of many of the alterations revealed by the intervention of the speculum."

After adducing a case illustrative of the errors of the "apostles of the speculum," and commenting upon it, the author proceeds:—"During the last two years that I have had the medical duty of the 'Hôpital de Lourcine,' I have examined upwards of 800 women labouring under diseases of the genital organs, of which number 600 have been or are now under treatment in my wards. I am therefore in a condition to give testimony on the subject, interesting alike in the highest degree to science and humanity."—"The neck of the uterus, examined by the speculum, differs in appearance in those who have not had children and with those who have been confined one or more times."

"In the first case, the cervix is round, slightly pyramidal, and nipple-like, it is pierced in its centre by a small circular orifice. This is what is called the virgin cervix. In the second case, the neck is more or less changed in form and flattened; it is more voluminous; a transverse fissure, more or less gaping, with some irregularity of border, has succeeded to the primitive circular perforation. This is called the maternal cervix. It has rarely happened to me to find the uterus totally free from leucorrhœa,* and only in young females.

"The vagina is naturally of a pale rose colour and rugous; the neck of the uterus is white, slightly tinged with rose colour. Many circumstances of course may change both the form and colour of the cervix, so as to lead us

* In explanation of this remarkable fact, regard must be had to the class of females coming under the author's examination.—*Trans.*

to suppose disease when none exists. Even in the same person an examination of the speculum has given different results if made at intervals, showing how utterly mistaken are those who attempt to decide upon the integrity of these organs after one examination." After pointing out some errors which have crept into much practice, but to which we are as yet strangers in this country, M. Gibert proceeds, "But to return to the principal subject of this memoir, there is a lesion of the os uteri, which merits special attention, although not of the grave nature which some persons have supposed. This is a species of ulceration which I have termed '*Granular erosion of the cervix uteri*,' ('*erosion granulée du col de l'uterus*,') and of which many examples are given in my manual of venereal diseases. This disease differs, however, from chancre properly so called. To give an idea of its frequency I may say, that out of 500 cases of disease, it was observed in 143. In some of them the erosion existed alone, without any other morbid symptom. With a few no leucorrhœa could be observed, and with others only a slight quantity of clear viscid mucus.

"With these exceptions (amounting to about fifteen) the patients affected with granular erosion presented more or less distinctly the other symptoms of syphilis.

"This granular erosion, which is rather superficial, is generally of a rounded form and circumscribed; it occupies sometimes the anterior, sometimes the posterior lip of the os uteri, occasionally both, and still more rarely it seems to penetrate the canal of the cervix. Its surface is granular and red, contrasting remarkably with the naturally smooth surface of the neck. It bleeds when slightly touched. The surface of the erosion is ordinarily concealed by a layer of thick, semi-transparent mucus, which it is somewhat difficult to remove.

"In the commencement this species of ulceration appears in the form of small granular points, slightly projecting, which soon excoriate and become confluent. It is very rare, however, for the ulceration to acquire any great extent.

"Though often connected with syphilitic symptoms it is impossible to attribute to them a venereal origin in all cases. Strictly speaking, the granular erosion is not a grave symptom. It neither gives rise to pain nor symptoms of metritis, as has been asserted. It may also be cured without having recourse to the *ordinary* energetic treatment. The cure, however, will generally take a considerable time. Cicatrization or stimulating applications do not appear to be of much use. More than once indeed the application of caustic has appeared injurious. The local remedy which has been most successful, is the spirituous tincture of nutgalls, or the "alcohol tannique," as described by M. Boutegny of Paris.

"Mixed with eight parts of water, and used as a vaginal injection, it dessicates the erosion and arrests the discharge.

"Topical remedies, however, cannot supersede the necessity of more general treatment. In most cases, mercurial preparations have been advisable, and of these the proto-ioduret has been preferred, given to the amount of one grain in the day as a pill, and continued from six weeks to two or three months. Its exhibition should be suspended, however, if the patient be threatened with salivation.

"The womb must be preserved from all local irritation, but it is by no means necessary for the patient to be confined to the horizontal position."

After this clear history of the disease in question, M. Gibert sums up the results of his observations in four propositions, with which we shall conclude.

1. It is inconvenient, useless, and dangerous (except in syphilis) to have recourse to the speculum uteri, unless their exist local symptoms indicative of organic lesion of the womb. Hysterical affections or abdominal pain are not to be deemed sufficient grounds for this investigation.

2. On the other hand, vaginal discharges, especially in females suffering from syphilis or suspected of it, demand an examination, by the 'toucher' and speculum, in order to a correct diagnosis. From this rule virgins or young girls are to be excepted.

3. Accidental redness of the neck of the uterus, aphthous patches and excoriations, slight ulceration, or granular erosion, are not of a very serious character, and never give rise to severe symptoms.

4. All these lesions may be cured without any local application. Yet in many cases the topical use of astringents (where the inflammation was neither too great nor too acute) has been decidedly useful. Caustics are rarely necessary, and when we have to employ them, the mildest and least painful should be chosen.—*Dublin Journal.*

---

The following combination of copaiva and cubebs, is stated in a late French inaugural thesis by M. Guisard, to be of certain power in the treatment of obstinate gonorrhœa:

℞. Balsam Copaib. }

Pulv. Cubeb. aa } ℥j.

Magnesiæ q. s.

Mft. et divid. in pil No. cxliv.

S. Take six in the morning, and six in the evening, for three or four days,—then eight, afterwards ten and twelve, gradually increasing the dose for three weeks, or even longer, according to the obstinacy of the discharge. The dose is large, and to that circumstance the efficacy of the treatment is probably due.

This formula is given by M. Bretonneau, of Tours, the author of the thesis: by giving the quantity for each in a single dose in the evening, this is digested more easily than if given in the morning.

